# Draft Genome Sequence of *Rhizobium* sp. Strain TBD182, an Antagonist of the Plant-Pathogenic Fungus *Fusarium oxysporum*, Isolated from a Novel Hydroponics System Using Organic Fertilizer

**DOI:** 10.1128/genomeA.00007-17

**Published:** 2017-03-16

**Authors:** Yuichiro Iida, Kazuki Fujiwara, Nobutaka Someya, Makoto Shinohara

**Affiliations:** a National Agriculture and Food Research Organization, Tsu City, Japan; b National Agriculture and Food Research Organization, Koshi City, Kumamoto, Japan; c National Agriculture and Food Research Organization, Tsukuba City, Japan

## Abstract

*Rhizobium* sp. strain TBD182, isolated from a novel hydroponics system, is an antagonistic bacterium that inhibits the mycelial growth of *Fusarium oxysporum* but does not eliminate the pathogen. We report the draft genome sequence of TBD182, which may contribute to elucidation of the molecular mechanisms of its fungistatic activity.

## GENOME ANNOUNCEMENT

Multiple parallel mineralization (MPM) is a novel hydroponics system in which soil microorganisms mineralize organic fertilizers in a hydroponic solution, supplying nitrate nitrogen to plants (1). This system allows coexistence of microbes and plant roots in the hydroponic solution, establishing a plant-microbe ecosystem in which microorganisms develop a biofilm community in the rhizosphere. Additionally, the MPM solution exhibits operative suppressiveness of both fungal and bacterial root-borne diseases, similar to natural disease-suppressive soils (2, 3). The complex interplay among biofilm-associated microorganisms can contribute to the development of suppressiveness to root-borne diseases in the MPM solution (4).

*Rhizobium* sp. strain TBD182 was isolated from the rhizosphere of tomato *Solanum lycopersicum* cultivated using the MPM hydroponics system as an antagonist of the root-borne pathogenic fungus *Fusarium oxysporum*, which causes serious damage in various plant species (4). TBD182 inhibits mycelial growth *in vitro* and chlamydospore germination of *F. oxysporum* but does not eliminate the pathogen (showing fungistatic activity) (4). Inoculation of TBD182 by itself showed virulence in *Arabidopsis thaliana* but did not cause tumor formation on tomato stems. To further characterize TBD182, we sequenced and annotated the entire genome sequence. The strain was deposited in the GenBank project in the Genetic Resource Center, the National Agriculture and Food Research Organization (accession no. MAFF720014).

Genomic DNA of TBD182 was isolated using the cetyltrimethylammonium bromide (CTAB) method, according to the standard protocol (5). The entire genomic sequence was determined using an Illumina HiSeq 2000 sequencing platform (Illumina, Hayward, CA, USA) with the paired-end approach, at the Beijing Genomics Institute. A total of 5,238,358 reads comprising 505 Mb (approximately 89-fold coverage) were organized into 35 scaffolds, with a scaffold *N*_50_ of 421 kb assembled using SOAPdenovo (version 2.04) (6, 7). The genome size was estimated to be 5.69 Mb by k-mer analysis. The presence of plasmids similar to pTi and pAt was detected. A total of 5,342 open reading frames were annotated using the Glimmer prediction (version 3.02) (8). All coding sequences were functionally annotated using BLAST (9) searches against the NCBI nonredundant (10), KEGG (11, 12), COG (13), and CAZy (14) databases.

Genome analysis revealed that TBD182 contained genes encoding type I, II, III, IV, and VI secretion systems and *N*-acyl-l-homoserine lactone synthesis and antibiotic synthesis components. Secretion systems may be associated with the fungistatic activity of TBD182. The type III secretion system (T3SS) in *Pseudomonas fluorescens* contributes to biocontrol activity against the plant-pathogenic oomycete *Pythium ultimum* (15), while *Burkholderia rhizoxinica* establishes symbiosis with the fungal phytopathogen* Rhizopus microsporus* through the T3SS (16). Additionally, enzymes potentially involved in the inhibition of fungal growth, including extracellular enzymes involved in the degradation of fungal cell walls, such as chitin-binding proteins, β-1,3 glucanases, a chitin deacetylase, and a chitosanase, metalloproteases, serine proteases, cysteine proteases, lipases, and phospholipases for degradation of membranes and proteins were found.

### Accession number(s).

The nucleotide sequence of the whole genome of TBD182 has been deposited at DDBJ/EMBL/GenBank under the accession number BDLN00000000. The version described in this paper is the first version, BDLN01000000.
